# Stabilizing Agents for Calibration in the Determination of Mercury Using Solid Sampling Electrothermal Atomic Absorption Spectrometry

**DOI:** 10.1100/2012/439875

**Published:** 2012-04-30

**Authors:** Hana Zelinková, Rostislav Červenka, Josef Komárek

**Affiliations:** Department of Chemistry, Faculty of Science, Masaryk University, Kotlářská 2, 61137 Brno, Czech Republic

## Abstract

Tetramethylene dithiocarbamate (TMDTC), diethyldithiocarbamate (DEDTC), and thiourea were investigated as stabilizing agents for calibration purposes in the determination of mercury using solid sampling electrothermal atomic absorption spectrometry (SS-ETAAS). These agents were used for complexation of mercury in calibration solutions and its thermal stabilization in a solid sampling platform. The calibration solutions had the form of methyl isobutyl ketone (MIBK) extracts or MIBK-methanol solutions with the TMDTC and DEDTC chelates and aqueous solutions with thiourea complexes. The best results were obtained for MIBK-methanol solutions in the presence of 2.5 g L^−1^ TMDTC. The surface of graphite platforms for solid sampling was modified with palladium or rhenium by using electrodeposition from a drop of solutions. The Re modifier is preferable due to a higher lifetime of platform coating. A new SS-ETAAS procedure using the direct sampling of solid samples into a platform with an Re modified graphite surface and the calibration against MIBK-methanol solutions in the presence of TMDTC is proposed for the determination of mercury content in solid environmental samples, such as soil and plants.

## 1. Introduction

Mercury and its compounds belong among the most toxic contaminants and have the ability to bioaccumulate. The main sources of mercury are volcanic activity, combustion of coal, and other human activities, through which mercury is released into water, soil, and sediments, whereby it enters into the food chain and causes health damages. Hence, the study of mercury content in environmental samples is very important [1–3].

For direct analysis of solid samples over the past years, solid sampling electrothermal atomic absorption spectrometry (SS-ETAAS) has been used. The solid samples are weighed on a graphite platform, which is inserted into a graphite tube. The advantages of this method are the use of a very small amount of sample and little sample pretreatment. The precision and accuracy of the results depend on the weighing process, distribution of particles in the sample, and its homogeneity. The disadvantages are increases in interferences and calibration technique [1, 2, 4–10].

In SS-ETAAS, the interferences, kinetic of atomization, shape of the signal, and sensitivity depend on the amount of the sample, the form of the analyte, and the matrix composition. If the properties of the sample and standards for calibration are different, an error can occur. Therefore, the right calibration technique is very important. The first method is the application of solid standards, such as certified reference materials with properties similar to the analyzed sample. The reference material is weighed on the graphite platform in various amounts, and every point of the calibration curve corresponds to one weight and measurement [11]. Vale et al. found that a higher amount of the sample has a depressive influence on the signal and distorts the calibration curve [5]. Maia et al. used five different reference materials and constructed the calibration curve as dependence of the normalized absorbance on the certified mercury content [11]. The disadvantage of calibration with solid standards is their low availability, high cost, limited concentration range, and limited possibility to prepare artificial samples [5, 6, 11]. If the matrix components interfere, the standard addition method may be used. This method is based on the assumption that a change in response for the sample and the sample with an addition of standard corresponds only to the change of the concentration. For solid samples two techniques can be used: the addition of an aqueous standard or the addition of reference material to the solid sample. A disadvantage of this method is that it is impossible to ensure the constant sample mass [7].

Another technique is calibration against aqueous standards. The main problem with the determination of mercury in a solution by ETAAS is the high volatility of the element and its compounds. Therefore, additions of thermal stabilizing agents are applied to avoid losses of mercury. Because inorganic mercury compounds are less volatile than the element itself, various oxidizing agents such as hydrogen peroxide, permanganate, or dichromate were used to prevent their reduction [1, 3, 8, 12, 13]. Reagents containing sulphur as dithizone, diethyldithiocarbamate (DEDTC), or tetramethylene dithiocarbamate (TMDTC) stabilize mercury by the formation of complex and subsequently mercury sulphide [1, 13–15]. A successful approach used to stabilize mercury is the application of modifiers to the graphite atomizer surface. Gold, platinum, palladium, rhodium, and iridium or their mixtures were investigated. Palladium is applied most frequently. The modifiers can be deposited onto an atomizer surface by the thermal or electrochemical method [1, 10–13]. In SS-ETAAS, calibration against aqueous standards was applied, utilizing oxidizing agents and modifiers of the graphite atomizer surface [1, 10]. In [1], a loss-free determination of mercury in aqueous calibration solutions was reached only through the addition of potassium permanganate and by using Pd, thermally deposited on the SS platform. This procedure was satisfactory for mercury determination in ash, sludge, and sediment reference materials. In our previous work [10], permanganate was used together with a Pd modifier, electrochemically deposited on the SS platform. However, the use of permanganate has some disadvantages. For technical reasons the dosing of only 3 *μ*L of KMnO_4_ solution onto the SS platform with concentration >10 g L^−1^ is possible. By the injection of a volume >3 *μ*L, a drop of solution with great viscosity is superimposed on the inner space of the SS platform and the insertion of the SS platform into to the graphite tube without any spills, using tweezers, is impossible. For the optimal total amount of KMnO_4_ (0.3 mg), a concentration of 100 g L^−1^ is required for 3 *μ*L of the solution. Moreover the preparation of such a solution of permanganate is difficult [10].

 Therefore, the aim of this work was to select another suitable stabilizing agent for calibration solutions. For this purpose, the influence of thiourea, tetramethylene dithiocarbamate, and diethyldithiocarbamate was studied. In our previous work [10], the electrodeposition from a drop of a modifier solution proved to be suitable method of preparing the Pd surface for the determination of mercury by SS-ETAAS. Because palladium has a relatively low boiling point, another metal for coating the SS platform was tested. On the basis of our previous results by the determination of gold with the complete electrochemical coating of the graphite tube surface, rhenium was chosen [16]. Palladium, and newly, rhenium were used as modifiers of the graphite platform surface for the determination of mercury in solid environmental samples as soil and plant.

## 2. Experimental

### 2.1. Instrumentation

A ZEEnit 650 atomic absorption spectrometer (Analytik Jena, Germany) with a transversely heated graphite tube and a solid sampling system SSA 61Z was used for all measurements. The spectrometer was equipped with a Zeeman-based and deuterium background corrector. The magnetic field of an electromagnet was applied to the graphite atomizer by the 2-field mode. Zeeman corrections were used throughout the work, and a deuterium device was used only in special cases. A mercury hollow cathode lamp at current 4.5 mA was used as the radiation source. Measurements were performed in the peak area mode at 253.7 nm using a spectral bandwidth of 0.5 nm. Calibration solutions were applied manually onto an SS graphite platform (Analytik Jena, Part no. 407-152.023) and introduced into the graphite tubes without a dosing hole (Analytik Jena, Part no. 407-152.316) in the same way as the solid samples. The calculated integrated absorbance per mg of the sample is introduced as the normalized absorbance. The temperature program for the determination of mercury is presented in Table 1.

 For comparison purposes, the mercury content in environmental materials was also determined using the AMA 254 analyzer (Altec, Czech Republic). The measurement in this single-purpose atomic absorption spectrometer is based on the combustion of a sample in a flow of oxygen and the subsequent capture of mercury by a gold amalgamator. After thermal release from amalgamator, the mercury vapour is measured. This pyrolysis approach in AAS is frequently used in analysis of environmental and biological materials, for example, marine sediments, soil, citrus and tomato leaves [17]. Each time, 40–100 mg of a sample was weighed or 10–200 *μ*L of solution was dosed in nickel boats. The solid samples were dried at 120°C for 60 s and decomposed at 650°C for 150 s. The AMA 254 analyzer was regularly calibrated using standard solutions of 1–1000 *μ*g·L^−1^ of mercury for the first (0–6 ng Hg) and second (0–200 ng Hg) calibration intervals. The calibration solutions were prepared by diluting the stock standard solution with 0.05% (m/v) K_2_Cr_2_O_7_ and 0.6% HNO_3_ to improve their stability. The accuracy of the results was controlled by analysis of the standard reference material GBW 07405. The relative standard deviation (RSD) was 3.2% (at 0.29 mg·kg^−1^ Hg, *n* = 10).

### 2.2. Chemicals and Solutions

Hg(II) solutions were prepared from the stock standard solution for mercury (1.000 ± 0.002 g L^−1^ Hg, Analytika, Czech Republic) in 2% HNO_3_ by dilution with 5% (v/v) HNO_3_. Thiourea p.a. (Sigma Aldrich), ammonium tetramethylene dithiocarbamate p.a. (TMDTC, Sigma Aldrich), acetic acid p.a. (Fluka), sodium acetate p.a. (Fluka), sodium diethyldithiocarbamate p.a., (DEDTC, Lachema), acetylacetone p.a. and methyl isobutyl ketone p.a. (MIBK, Lachema) were used for the preparation of calibration solutions. A stock solution of 110 g L^−1^ KMnO_4_ (Merck) was prepared as in [10] with the support of an ultrasonic bath and added to the calibration solutions for a final concentration of 100 g L^−1^. PdCl_2_ (Merck, Darmstadt, Germany) and NH_4_ReO_4_ (Analytika, Czech Republic) standard solutions containing 10 g L^−1^ Pd or Re were used to modify the graphite platform surface.

### 2.3. Samples and Their Treatment

 A Certified reference material (CRM) soil GBW 07405 (National Centre for Standard Materials, Beijing, China) and the environmental samples of soil II and plant *Scirpus* from the Hg-polluted area were used. The environmental samples were ground in a mill Fritsch Pulverisette 7 with balls from Si_3_N_4_ and passed through a nylon sieve for a particle size of ≤56 *μ*m. The aliquots of environmental samples between 0.1 and 0.5 mg or CRM GBW 07405 2–10 mg were weighed directly onto the SS platforms and inserted into a graphite tube. Before each weighing on the SS platform, these ground samples were carefully stirred. The residues of solid samples after atomization were easily removed from the platform.

### 2.4. Electrodeposition of Palladium and Rhenium from a Drop of Solutions

**Figure 1:**
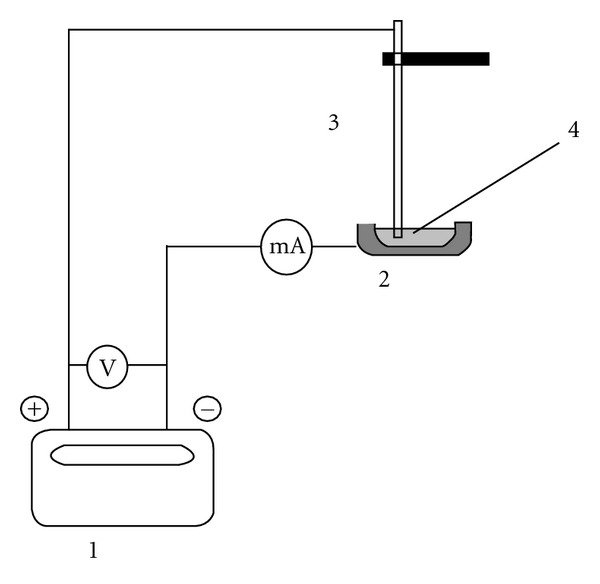
Schematic diagram for electrodeposition from a drop. (1) Power supply, (2) SS platform: cathode, (3) Pt wire: anode, (4) drop of modifier solution.

The surface modifiers were applied to the graphite platform using 7 injections of 20 *μ*L of solution of 2 g L^−1^ Pd or Re. The graphite platform with a drop of modifier solution served as the cathode and a Pt wire was used as the anode (Figure 1). Electrodeposition of every drop proceeded by the current 10 mA for 5 min. After each deposition, the surface of the SS platform was rinsed with water, dried, the SS platform was inserted into the graphite tube, and the temperature program started according to Table 2. The amount of Pd or Re electrodeposited onto the SS platform was calculated from the difference of its content in the solution before and after electrolysis. During electrodeposition 250 *μ*g Pd or Re was deposited.

### 2.5. Preparation of Calibration Solutions of Hg(II) in the Presence of TMDTC and DEDTC

 Calibration solutions of Hg(II) in the presence of TMDTC and DEDTC were prepared using two methods:

- The extraction of mercury with chelating agents into MIBK or acetylacetone. 1 mL of mercury(II) solution, 2 mL of 2.5 g L^−1^ TMDTC or DEDTC aqueous solution and 1 mL of acetate buffer (pH 5) were pipetted into the extraction tube. After shaking, 4 mL acetylacetone or MIBK was added. The chelates were extracted into the organic phase on the shaker at a speed of 300 RPM for 1 h. The extraction efficiency was checked by measuring the absorbance of the aqueous phase.
- The preparation of MIBK-methanol solution from an aqueous methanol solution refilled by MIBK.The calibration solutions of Hg(II) in the presence of TMDTC were prepared from 0.5 mL of Hg(II) aqueous solution, 1 mL of 25 g L^−1^ TMDTC in methanol and 1 mL of 1 mol L^−1^ sodium acetate in methanol. After shaking, the solution was diluted with MIBK to 10 mL to formation of single phase.

## 3. Results and Discussion

### 3.1. Stability of Surface Modifiers

The modifier of the graphite platform surface has a limited lifetime. To its investigation, the solution of a constant concentration of mercury was always applied after the 10 atomization cycles, and mercury absorbance was measured. In our previous work [10], the lifetime for the Pd modifier was found to be 100–120 atomization cycles. In case of the Re, a sensitivity decrease of 10% was observed after 200 cycles. The Re modifier is more stable due to a higher boiling point than Pd. The surface of the platforms was always recoated with optimal mass of 250 *μ*g Pd or Re after 100 or 200 cycles. By using the less mass of modifier, lower sensitivity was observed. The electrodeposition from a drop proved to be a suitable way for graphite surface modification with rhenium as well. This technique does not require a special cell, the electrolysis spans a short time (35 min), and the electrochemical coating of the SS platform is ensured.

### 3.2. Stabilizing Agents for Hg(II) in Solution

**Figure 2:**
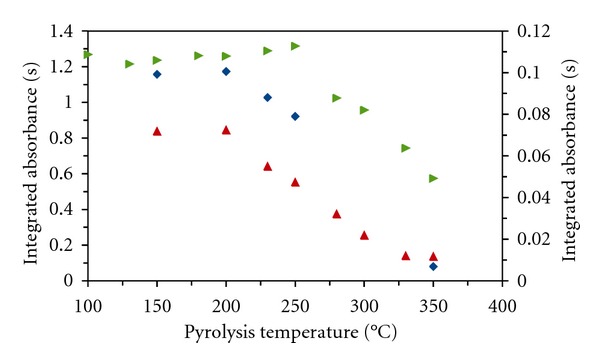
Pyrolytic curves for Hg(II) solutions in the presence of stabilizing agents and Pd surface modifier. left axis: ▲ DEDTC, *◆* TMDTC; right axis: ▸ without stabilizing agent.

**Figure 3:**
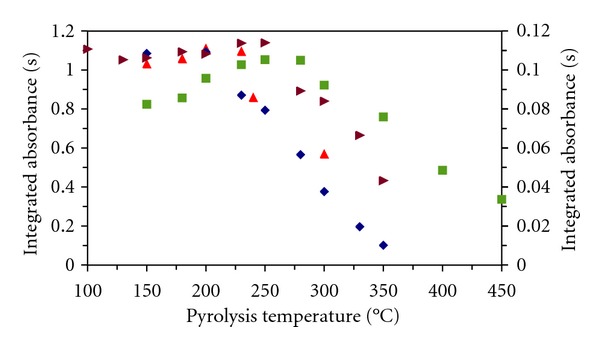
Pyrolytic curves for Hg(II) solutions in the presence of stabilizing agents and Re surface modifier. left axis: ■ thiourea (deuterium background correction), ▲ DEDTC, *◆* TMDTC; right axis: ▸ without stabilizing agent.

The pyrolysis curves (Figures 2 and 3) and the influence of the amounts of stabilizing agents on mercury absorbance were investigated for both surface modifiers and all stabilizing agents. The solutions were injected in a volume 20 *μ*L. For comparison the results obtained for solutions of mercury(II) only in diluted nitric acid without a stabilizing agent are shown. The integrated absorbance for mercury is low and indicates that part of mercury was lost, probably already during the drying stage. The investigated graphite surface modifiers thus have little stabilizing effect for mercury in a diluted nitric acid solution during the drying stage. Therefore, the addition of a stabilizing agent into calibration solutions is necessary. Maximum usable pyrolysis temperatures for solutions of mercury(II) with stabilizing agents are shown in Table 3. In this table, the data for potassium permanganate with Pd modifier [10] and newly measured with Re modifier are mentioned.

The aqueous calibration solutions in the presence of 1 g L^−1^ thiourea were prepared at pH 1.5. With Zeeman background correction for both Pd and Re modifiers, an overcorrection of the signal was observed and the absorbance record was made impossible. A change of pH in range 1.5–5, similarly to the concentration of thiourea in range 0.1–30 g L^−1^, did not eliminate this effect. With deuterium background correction and Pd modifier, a dual-split peak was observed. By using the graphite platform, modified with Re and with deuterium background correction, the determination of mercury was possible with RSD = 3.5–4.1% for 5–10 ng Hg (*n* = 5). The calibration curve was linear to 10 ng Hg (*R* ^2^ = 0.9955).

TMDTC forms with Hg(II) stable chelate, which may be extracted in an organic solvent. Acetylacetone and MIBK were selected for this purpose. The use of an acetylacetone as a solvent was not appropriate, because overcorrection of the signal was observed. Preevaporation of acetylacetone under an infralamp did not eliminate this effect. By using MIBK as a solvent, mercury was stabilized to 200°C for both surface modifiers. However, the overcorrection of the signal was observed with the use of a Pd modifier and Zeeman background correction at pyrolysis temperatures 260–340°C. This effect was not observed by using deuterium background correction. The presence of 0.01–4 mg TMDTC in 20 *μ*L of calibration solution had no influence on the mercury signal, and the amount of 0.05 mg TMDTC was chosen as optimal. Both methods of preparation of the calibration solutions, with the use of extract in MIBK and with aqueous methanol solution refilled by MIBK, yielded the same results. The RSD values for the measurements with extracts by pyrolysis temperature 200°C were 1.3–2.4% for 10–15 ng Hg (*n* = 5) with Pd modifier and 1.9-2.0% for 10–15 ng Hg (*n* = 5) with Re modifier. The RSD of mercury determination in MIBK-methanol solution was 1.9–2.4 for 10–15 ng Hg (*n* = 5) with Pd modifier and 2.0–2.5% for 10–15 ng Hg (*n* = 5) with Re modifier. Calibration curves were linear to 15 ng Hg (*R* ^2^ = 0.9975 or *R* ^2^ = 0.9994  for extracts with Pd or Re modifier and *R* ^2^ = 0.9976 or *R* ^2^ = 0.9994  for MIBK-methanol solution with Pd or Re modifier). By using the Pd modifier, the detection limit, 128 pg Hg, acquired through 6 repetitive firings of the platform with TMDTC enables the determination of 0.43 mg kg^−1^ Hg for an optimum sample mass of 0.3 mg. By using the Re modifier, the detection limit was 120 pg Hg and 0.40 mg kg^−1^ Hg.

DEDTC as the chelating agent was not suitable for stabilization of mercury in calibration solutions, because dual-split peaks were obtained for both types of modifiers and background corrections. The change of the concentration of DEDTC in a range of 0.01–4 mg DEDTC in 20 *μ*L of solution or modification of the temperature program had no influence on the shape of peak.

The use of 100 g L^−1^ of permanganate in combination with the Re modifier of the graphite platform surface provided better results than those in combination with Pd modifier. The disadvantage of permanganate is the necessity of dosing only 3 *μ*L of KMnO_4_ solution and difficulty in preparing the stock solution of permanganate. The RSD of mercury determination was 4.0–4.9% for 10–15 ng Hg (*n* = 5) with the Pd modifier [10] and 3.5–5.0% for 10–15 ng Hg (*n* = 5) with the Re modifier. Calibration curves were linear to 15 ng Hg (*R* ^2^ = 0.9991  for the Pd modifier and *R* ^2^ = 0.9998  for Re modifier). By using the Pd modifier, the detection limit, 120 pg Hg, was acquired from 10 repetitive firings of platform with KMnO_4_ and enabled determination of 0.40 mg kg^−1^ Hg for an optimum sample mass of 0.3 mg. By using the Re modifier, the detection limit was 110 pg Hg and 0.37 mg kg^−1^ Hg.

### 3.3. Analytical Results

**Figure 4:**
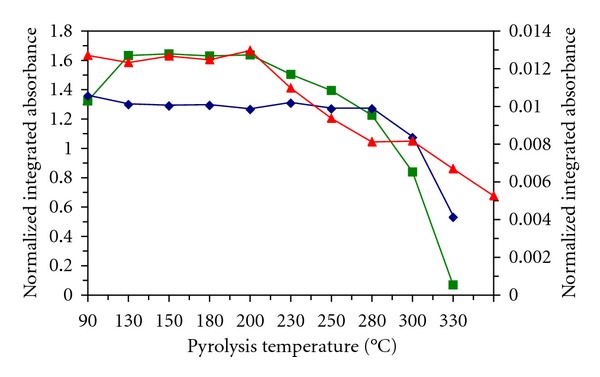
Pyrolytic curves for solid samples in presence Pd surface modifier. left axis: ■ plant, *◆* soil II; right axis: ▲ CRM CBW 07405.

**Figure 5:**
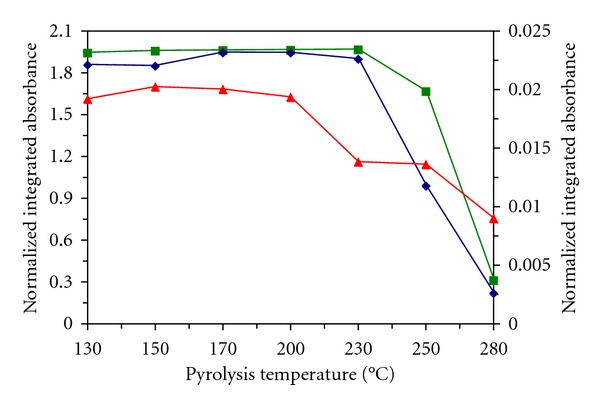
Pyrolytic curves for solid samples in presence Re surface modifier. left axis: ■ plant, *◆* soil II; right axis: ▲ CRM CBW 07405.

The pyrolytic curves (Figures 4 and 5) were investigated for solid samples by using both surface modifiers and from their shape maximum usable pyrolysis temperature results for soil II 280°C with Pd or 230°C with Re, for plant 200°C with Pd or 230°C with Re, and for CRM GBW 07405 200°C with both surface modifiers. The results obtained for mercury content in soil II, plant, and CRM GBW 07405 using SS platforms modified with palladium or rhenium electrolytic from a drop of modifier solutions and calibration against MIBK extracts or MIBK-methanol solutions in presence of TMDTC are given in Table 4. For comparison the results obtained for permanganate with the Pd modifier presented in [10] or with the Re modifier newly measured in this paper are also mentioned. In all cases, the results are in good agreement with the certified value and with those obtained by measurement on the AMA 254 analyzer. The RSD values are dependent on the number of platform firings and consequently on the state of the platform surface. According to the results, the switch from the calibration solutions to the samples on the modified surface can also play a certain role. It can be connected with increased precision of mercury determination using calibration solutions in the presence of TMDTC.

## 4. Conclusion

 Agents such as TMDTC, DEDTC, and thiourea were investigated for complexation of mercury in calibration solutions and its thermal stabilization in a solid sampling platform in the determination of mercury using SS-ETAAS. The calibration solutions were used in the form of MIBK extracts or MIBK-methanol solutions for the TMDTC and DEDTC chelates and aqueous solutions for the thiourea complexes. Only calibration with TMDTC was successful. MIBK-methanol solutions in the presence of TMDTC are easier to prepare than MIBK extracts. Therefore, calibration with MIBK-methanol solutions in the presence of TMDTC was preferred. Higher precision in calibration and easier manipulation with the solutions makes the calibration with MIBK-methanol solutions preferable to the heretofore used potassium permanganate [1, 10]. The use of standard solutions for calibration also provides the best precision and lowest uncertainty prior to the use of reference materials. The surface of the graphite platforms for solid sampling was modified with palladium or rhenium using electrodeposition from a drop of solutions. This process of electrochemical coating of the SS platform surface is promising for the preparation of graphite surface modifiers in SS-ETAAS. The Re modifier is preferable due to the higher lifetime of platform coating. The use of the SS-ETAAS method with a modified surface of the SS platform and calibration against stabilized calibration solutions reduces the time of analysis compared with the mercury determination after the sample digestion. Sample preparation requires only routine grinding and homogenization. The new SS-ETAAS procedure using direct sampling solid samples into a platform with an Re modified graphite surface, and the calibration against MIBK-methanol solutions in the presence of TMDTC is proposed for the determination of mercury content in solid environmental samples, such as soil and plants. With calibration against MIBK-methanol solutions in the presence of TMDTC, the detection limit was 120 pg and with a sample mass of 0.3 mg it was 0.4 mg kg^−1^ Hg.

## Figures and Tables

**Table 1:** Temperature program for the determination of mercury.

Stage	Temperature (°C)	Ramp (°C s^−1^)	Hold (s)
Drying	90^a^, 120^b^	30	15
Pyrolysis	200	30	40
AZ^c^	200	0	6
Atomization^d^	1100	1500	10
Cleaning	1700	200	4

^a^Drying temperature for aqueous solutions, ^b^drying temperature for MIBK calibration solutions in the presence of TMDTC and DEDTC, ^c^auto zero, ^d^gas stop.

**Table 2:** Temperature program (according to study [1]) for treatment of platform after the electrodeposition of modifiers from a drop of solutions.

Stage	Temperature (°C)	Ramp (°C s^−1^)	Hold (s)
Drying	90	30	15
Pyrolysis	250	20	35
AZ^a^	250	0	6
Atomization	1000	1000	10
Cleaning	2000	200	5

^a^Auto zero.

**Table 3:** Maximum pyrolysis temperatures for the determination of mercury in calibration solutions with the stabilizing agents.

	Temperature/°C	
Agents	Pd	Re
Potassium permanganate	250^a^	280
Thiourea	—	280
TMDTC	200	200
DEDTC	200	230

^a^[10].

**Table 4:** Results obtained for mercury content in environmental materials using SS-ETAAS with modification of platform surface and calibration against calibration solutions.

Material	Obtained value ± SD/mg kg^−1^ (*n* = 5)						Certified and determined value ± SD/mg kg^−1^
	TMDTC/MIBK		TMDTC/methanol/MIBK		KMnO_4_^a^		
	Pd	Re	Pd	Re	Pd^a^	Re	
Soil II	37.2 ± 1.8	37.7 ± 1.9	38.9 ± 1.3	38.3 ± 1.4	38.8 ± 1.6	36.6 ± 2.1	38.1 ± 0.7
Plant	35.7 ± 1.7	37.5 ± 1.8	37.4 ± 1.6	37.7 ± 1.4	37.7 ± 2.0	36.5 ± 1.8	37.6 ± 0.5
GBW 07405	0.25 ± 0.02	0.27 ± 0.02	0.30 ± 0.02	0.29 ± 0.02	0.30 ± 0.01	0.27 ± 0.02	0.29 ± 0.03

^a^According to procedure in [10].

## References

[1] da Silva AF, Welz B, Curtius AJ (2002). Noble metals as permanent chemical modifiers for the determination of mercury in environmental reference materials using solid sampling graphite furnace atomic absorption spectrometry and calibration against aqueous standards. *Spectrochimica Acta—Part B*.

[2] Grobecker KH, Detcheva A (2006). Validation of mercury determination by solid sampling Zeeman atomic absorption spectrometry and a specially designed furnace. *Talanta*.

[3] López-García I, Sánchez-Merlos M, Hernández-Córdoba M (1997). Determination of mercury in soils and sediments by graphite furnace atomic absorption spectrometry with slurry sampling. *Spectrochimica Acta—Part B*.

[4] Welz B (1999). Atomic absorption spectrometry—pregnant again after 45 years. *Spectrochimica acta, Part B*.

[5] Vale MGR, Silva MM, Welz B, Lima ÉC (2001). Determination of cadmium, copper and lead in mineral coal using solid sampling graphite furnace atomic absorption spectrometry. *Spectrochimica Acta—Part B*.

[6] Coşkun N, Akman S (2004). Direct determination of manganese in vitamin-mineral tablets using solid sampling electrothermal atomic absorption spectrometry. *Talanta*.

[7] Resano M, García-Ruiz E, Aramendía M, Belarra MA (2005). Solid sampling-graphite furnace atomic absorption spectrometry for Hg monitoring in soils. Performance as a quantitative and as a screening method. *Journal of Analytical Atomic Spectrometry*.

[8] da Silva AF, Lepri FG, Borges DLG, Welz B, Curtius AJ, Heitmann U (2006). Determination of mercury in biological samples using solid sampling high—resolution continuum source electro thermal atomizaion atomic absorption spectrometry with calibration againts aqueous standards. *Journal of Analytical Atomic Spectrometry*.

[9] Detcheva A, Grobecker KH (2006). Determination of Hg, Cd, Mn, Pb and Sn in seafood by solid sampling Zeeman atomic absorption spectrometry. *Spectrochimica Acta—Part B*.

[10] Červenka R, Zelinková H, Konečná M, Komárek J (2010). Electrochemical modification of a graphite platform for a solid sampling electrothermal atomic absorption spectrometry of mercury. *Analytical Sciences*.

[11] Maia SM, Welz B, Ganzarolli E, Curtius AJ (2002). Feasibility of eliminating interferences in graphite furnace atomic absorption spectrometry using analyte transfer to the permanently modified graphite tube surface. *Spectrochimica Acta—Part B*.

[12] Baralkiewicz D, Gramowska H, Kózka M, Kanecka A (2005). Determination of mercury in sewage sludge by direct slurry sampling graphite furnace atomic absorption spectrometry. *Spectrochimica Acta—Part B*.

[13] Bulska E, Kandler W, Hulanicki A (1996). Noble metals as permanent modifiers for the determination of mercury by electrothermal atomic absorption spectrometry. *Spectrochimica Acta—Part B*.

[14] Kunert I, Komárek J, Sommer L (1979). Determination of mercury by atomic absorption spectrometry with cold vapour and electrothermal techniques. *Analytica Chimica Acta*.

[15] Grigič I, Hudnik V, Gomišček S (1989). Behaviour of mercury complexes in a graphite tube furnace for atomic absorption spectrometry. *Analytica Chimica Acta*.

[16] Konečná M, Komárek J (2007). Utilization of electrodeposition for electrothermal atomic absorption spectrometry determination of gold. *Spectrochimica Acta—Part B*.

[17] Costley CT, Mossop KF, Dean JR, Garden LM, Marshall J, Carroll J (2000). Determination of mercury in environmental and biological samples using pyrolysis atomic absorption spectrometry with gold amalgamation. *Analytica Chimica Acta*.

